# ADHD presenting as recurrent epistaxis: a case report

**DOI:** 10.1186/1753-2000-5-13

**Published:** 2011-04-24

**Authors:** Yasir H Rather, Ajaz A Sheikh, Aalia R Sufi, Ateeq A Qureshi, Zaid A Wani, Tasneem S Shaukat

**Affiliations:** 1 Department of Psychiatry, Government Psychiatric Diseases Hospital, GMC Srinagar, Kashmir, J&K, 190010, India; 2 Department of Ophthalmology, Government Medical College, Srinagar, Kashmir, J&K, 190001, India; 3 Child and Family Consultation Service Thorpe Coombe Hospital, Shernhall Street, Walthamstow, London. E17 3EA, UK

## Abstract

Epistaxis is an important otorhinolaryngological emergency, which usually has an apparent etiology, frequently local trauma in children. Here we present a case report wherein the epistaxis was recalcitrant, and proved to have a psychiatric disorder as an underlying basis. The child was diagnosed with Attention Deficit/Hyperactivity Disorder, hyperactive type, which led to trauma to nasal mucosa due to frequent and uncontrolled nose picking. Treatment with atomoxetine controlled the patient's symptoms and led to a remission of epistaxis.

## Background

### Introduction

Children commonly present with nosebleed and these episodes are rarely life threatening. The majority of nosebleeds are mild, spontaneous and self-limited. However some children suffer from repeated nosebleeds or, to use its clinical name, 'recurrent idiopathic epistaxis'. These nosebleeds often cause significant parental concerns and remain a challenging problem to patients and physicians alike [1,2].

Initiating factors include local inflammation, mucosal drying, and local trauma (including nose picking). Most of the studies have emphasized the fact that there are no apparent causes in habitual nose bleeders. However, there may be some underappreciated factors that place many children at risk for injury [3]. One of these factors may be the presence of attention-deficit/hyperactivity disorder (ADHD), which is now believed to be the most common neurobehavioral disorder in children[4]. The purpose of this case report is to describe the case of the child with a diagnosis of ADHD who suffered severe recurrent epistaxis, and to highlight the possible importance of this co-morbidity and its treatment in the context of paediatric trauma.

## Case presentation

### History

A 12 year old boy presented with a 2 month history of recurrent epistaxis to the emergency department for his fourth episode. The first episode had occurred 2 months back and was treated by local pressure and a haemostatic drug. The second episode occurred 2 weeks later and was treated similarly. A week later, the patient had another bout of nose bleed, heavier this time, which had to be treated with an anterior nasal pack, and silver nitrate cauterization of the wound later on. The current episode was from the same site and needed nasal packing again.

On all occasions there was no history of an apparent physical trauma to the nose, nor were there any symptoms to suggest an upper respiratory infection or allergic rhinitis. There was no bleeding from any other site in the body. The patient was not using any medicines. The patient was not suffering from any diagnosed medical condition. There was no family history of a similar illness.

### Physical Examination

On arrival the patient was awake, alert and fully oriented. He was bleeding moderately from left nostril. On physical examination his vital signs were stable. ENT examination showed active bleeding from left anterior nares. Rest of physical examination was normal. All through the examination, the child acted fussy and had difficulty remaining focused on a given task. He continuously rocked and fidgeted in the examination chair. Even frequent reprimanding couldn't discipline the child. This prompted the attending resident to seek a psychiatric consultation.

### Psychiatric Screening

#### History

A detailed evaluation revealed a child who had no problems in preschool. In kindergarten, he seemed to learn alphabets and numbers normally. The parents had noticed that he seemed more disorganized and inattentive than his older brother was at the same age. They often had to repeat instructions, and he left tasks half-finished. In primary school the patient had mild difficulty with mathematics, and the teacher use to be concerned about his not listening much of the time. The patients' school work was inconsistent and he often failed to finish his assignments. The parents also admitted a frequent nose picking behavior of the patient, which they couldn't correct with even punitive methods.

#### Mental Status Examination

When the patient was seen in the child and adolescent psychiatry department, he appeared as an attractive teenager who looked his stated age and was of average build but he showed grossly conspicuous behaviour. During interview he constantly shifted position, folded arms behind his head or leaned over the table in front of him and at times fiddled with his nose. He also got out of his seat frequently, played with buttons on clothes and couldn't sit still. His attitude was over familiar, pushy, demanding and lacking distance. He showed difficulty in sustaining attention and concentration which was elicited in writing and reading task given to him in interview. He was oriented in time, place and person. Intelligence was normal

#### Diagnostic Inventory

A diagnosis of Attention Deficit/Hyperactivity Disorder, hyperactive type was suggested.

## Investigation

The patients hemoglobin was decreased at 10.2 gm/dl (11-13 gm/dl), platelet count was normal at 230,000 per microliter (150,000 to 400,000 per microliter). The coagulation profile was normal. TLC & DLC, ESR, RBC indexes were normal. Serum chemistry, TFT, urine exam and X-ray chest were also normal. ECG only showed sinus tachycardia (HR: 108/min).

### Management and Course

The patient was started on atomoxetine at 9 mg/bd, and weekly behavioral therapy sessions (including habit-reversal therapy), aimed at decreasing the nose picking behavior. The dose of atomoxetine was raised two weeks later to 18 mg/bd (calculated @ 0.5 mg/kg/d), while the behavior therapy was continued. The patient was sent for ENT follow-up as well, who after evaluation referred the patient the back, with no alteration in the treatment. The patient was followed up at weekly intervals. At 4 (Four) weeks, the patient's hyperactive behavior, including nose picking, was much controlled. ENT checkup confirmed healing of the nasal wound. The drug treatment was continued at the same dose and patient continues to follow up on a monthly basis with no further episode of nosebleed.

## Discussion

ADHD is one of the commonest behaviour disorders in children and has a known prevalence of around 5% in general population. It is characterised by hyperactivity, inattention and impulsiveness that is inappropriate to age and occurring in several different setting [4].

The male to female ratio is about 9:1 in mental health clinic samples in contrast to 2:1 in epidemiologically samples. Females present more often with less disruptive symptoms while boys present with more disruptive behaviour leading to clinician referral [5]. The quality of the activity has often been described as disruptive and purposeless [6].

To our knowledge the hyperactivity nature of ADHD has never been reported as a risk/associative factor for repeated nose picking trauma. The psychiatric literature however has recognised that due to underlying hyperactivity and impulsiveness and inattention, these children have proneness towards injury [7]. In addition there are studies which show possible association between self insertion of nasal and aural foreign bodies and ADHD [8].

We report a case of a child who was treated for recurrent idiopathic epistaxis and in whom we diagnosed associated ADHD with history of nose-picking behaviour. It was noticed that as soon as treatment was started for management of ADHD, his hyperactive behaviour came under control and so did his nose-picking behaviour. Although the child was receiving behavioural therapy as well but the dramatic response in the nose-picking behaviour in parallel with the hyperactive behaviour made us to presume that our case of recurrent epistaxis might be due to repeated nose picking attributable to underlying hyperactivity and fidgetiness. One explanation is that nose picking might function as tension reduction behaviour, this tension and anxiety may be secondary to ADHD and its consequences.

We used Child Behaviour Checklists (CBCL) as an aid to rule out other co-morbidity like OCD, Tic disorder and SIB [9]. Moreover since ADHD treatment does not treat OCD and OCD treatments do not treat ADHD, children with both disorders will likely need combined pharmacotherapy. Both these disorders are responsive to very different pharmacological interventions, serotonin reuptake inhibitors are the mainstay of pharmacological treatment for OCD, the treatment of one disorder without parallel treatment of the other will leave children with the co-morbid condition inadequately treated. But in our case there was global improvement in symptoms due to atomoxetine so it can be presumed that the nose picking was associated to ADHD [10].

The other differential diagnosis in our patient would be rhinotillexomania which is a benign habit in children, but because of other associated features of hyperactivity and inattentiveness that was ruled out [11]. In addition, other causes like rhinitis, systemic disease, or remotely cocaine abuse were all sequentially excluded.

Since children with ADHD may be at an increased risk for recurrent epistaxis following recommendation may be made for the ENT specialist. They should look for symptoms of ADHD when evaluating any child with recurrent epistaxis and nose picking behaviour or other associated nose injury. Early recognition of symptoms of ADHD in this setting may allow for referral of a potential injury prone child to a paediatric psychiatrist before a more serious or life threatening injury occurs.

## Conclusion

In conclusion, we offer this uncommon case to demonstrate possibility of childhood attention deficit/hyperactivity disorder underlying self-inflicted digital nasal trauma in children. The possibility of self-inflicted injuries of such nature should be considered whenever encountering cases of epistaxis in children.

## Consent

Written informed consent was obtained from the parents of patient for publication of this Case Report. A copy of the written consent is available for review by the Editor-in-Chief of this journal.

## Competing interests

The authors declare that they have no competing interests.

## Authors' contributions

YHR conceptualized the manuscript idea, participated in the literature review, and prepared the first draft of the manuscript. AS performed the psychiatric assessment of the patient including the follow-up. ARS and AAQ made substantive contributions to the conceptualization of the manuscript and contributed to the literature review. TS and ZAW contributed in revising the manuscript for important intellectual content. All authors read and approved the final manuscript.
